# Genome-Wide Association Study of Breast Cancer in the Japanese Population

**DOI:** 10.1371/journal.pone.0076463

**Published:** 2013-10-15

**Authors:** Siew-Kee Low, Atsushi Takahashi, Kyota Ashikawa, Johji Inazawa, Yoshio Miki, Michiaki Kubo, Yusuke Nakamura, Toyomasa Katagiri

**Affiliations:** 1 Laboratory for Statistical Analysis, Center for Integrative Medical Sciences, The Institute of Physical and Chemical Research (RIKEN), Yokohama, Japan; 2 Laboratory of Molecular Medicine, Human Genome Center, Institute of Medical Science, The University of Tokyo, Tokyo, Japan; 3 Laboratory for Genotyping Development, Center for Integrative Medical Sciences, The Institute of Physical and Chemical Research (RIKEN), Yokohama, Japan; 4 Department of Molecular Cytogenetics, Medical Research Institute and School of Biomedical Science, Tokyo Medical and Dental University, Tokyo, Japan; 5 Department of Genetic iagnosis, The Cancer Institute, Japanese Foundation for Cancer Research, Tokyo, Japan; 6 Department of Medicine and Surgery, The University of Chicago, Chicago, Illinois, United States of America; 7 Division of Genome Medicine, Institute for Genome Research, The University of Tokushima, Japan; MOE Key Laboratory of Environment and Health, School of Public Health, Tongji Medical College, Huazhong University of Science and Technology, China

## Abstract

Breast cancer is the most common malignancy among women in worldwide including Japan. Several studies have identified common genetic variants to be associated with the risk of breast cancer. Due to the complex linkage disequilibrium structure and various environmental exposures in different populations, it is essential to identify variants associated with breast cancer in each population, which subsequently facilitate the better understanding of mammary carcinogenesis. In this study, we conducted a genome-wide association study (GWAS) as well as whole-genome imputation with 2,642 cases and 2,099 unaffected female controls. We further examined 13 suggestive loci (*P*<1.0×10^−5^) using an independent sample set of 2,885 cases and 3,395 controls and successfully validated two previously-reported loci, rs2981578 (combined *P*-value of 1.31×10^−12^, OR = 1.23; 95% CI = 1.16–.30) on chromosome 10q26 (*FGFR2*), rs3803662 (combined *P*-value of 2.79×10^−11^, OR = 1.21; 95% CI = 1.15–.28) and rs12922061 (combined *P*-value of 3.97×10^−10^, OR = 1.23; 95% CI = 1.15–.31) on chromosome 16q12 (*TOX3-LOC643714*). Weighted genetic risk score on the basis of three significantly associated variants and two previously reported breast cancer associated loci in East Asian population revealed that individuals who carry the most risk alleles in category 5 have 2.2 times higher risk of developing breast cancer in the Japanese population than those who carry the least risk alleles in reference category 1. Although we could not identify additional loci associated with breast cancer, our study utilized one of the largest sample sizes reported to date, and provided genetic status that represent the Japanese population. Further local and international collaborative study is essential to identify additional genetic variants that could lead to a better, accurate prediction for breast cancer.

## Introduction

Breast cancer is the most common malignancy among women worldwide. In Japan, breast cancer comprises approximately 19% of all female cancers; it is the fifth leading cause of cancer death among women with an estimated death of 12,731 in 2011. Its incidence is about 86.0 cases/100,000 individuals/year and 56,289 were newly diagnosed to have breast cancer in 2007 (http://ganjoho.jp/data/public/statistics/backnumber/2012/files/cancer_statistics_2012.pdf). Even though the 5-year survival rate for breast cancer is relatively better compared to other malignancies, the age-adjusted incidence and mortality rate for breast cancer has revealed a significant increase since 1970s in Japan. Hence, breast cancer is one of the most important medical issues to be addressed. In particular, individual risk assessment (genetic and environmental factors), early detection by biomarkers and mammography screening are critically important to reduce breast cancer-associated death.

Although risk factors such as age, age at menarche, ethnicity, reproductive and menstrual history, oral contraceptives, hormone therapy, radiation exposure, mammographic breast density, alcohol intake, dietary folate intake, physical activity and benign breast diseases have been reported [1]–[7], it is well known that breast cancer is a complex polygenic disease in which genetic factors play an important role in disease etiology and pathogenesis. Individuals who have first-degree relatives with breast cancer are indicated to have approximately 2.1-fold higher risk for the disease [8]. Previous linkage analysis identified mutations in two highly-penetrant genes, *BRCA1* and *BRCA2*, as one of the major cause of inherited cancer in many families [9], [10]. In addition, mutations in *ATM*, *TP53*, *CHEK2*, *PTEN*, *CDH1*, *STK11* and *PALB2* genes also confer risk to breast cancer [11]–[17]. Nevertheless, mutations in these genes are not common in the general population and account for 5–0% of breast cancer cases. Hence, there is likely to be other genetic variants that contribute to the etiology of this cancer. Since 2007, approximately 67 common genetic variants have been identified to be associated with breast cancer through genome-wide association studies (GWAS) and international collaborative study from European and Asian descendants [18]–[34]. Due to the complex linkage disequilibrium, differences in allele frequencies and environmental exposure in different populations, it is of importance to identify common genetic variants associated with breast cancer in specific populations, which subsequently facilitate the development of useful prediction systems. Our group has previously reported a GWAS to identify common genetic variants associated with hormonal receptor positive breast cancer [35], but the current study has increased the sample size, which is one of the biggest sample size that represent the Japanese population, and has used a genotyping panel with better coverage aiming to identify common genetic variants associated with all types of breast cancer. Furthermore, we also evaluated the association of previously-identified loci showing the association with breast cancer in the European population and East Asian in the current dataset. Lastly, we conducted whole-genome imputation by referring to 1000G reference panel to increase the coverage of this GWAS study.

## Subject and Methods

### Study population

We recruited all DNA samples from the Biobank Japan Project (http://biobankjp.org). The Biobank Japan is a bank that has collected DNAs and serum of nearly 200,000 individuals, who had been diagnosed to have one or more of 47 common diseases including various types of cancer from 66 collaborating hospitals in Japan. One of the major objectives of this project is to identify genetic variants that are associated with common diseases and to identify individuals who are at risk for various diseases. In this study, we selected a total of 5,610 breast cancer patients who had been registered in the Biobank Japan. We subsequently divided these patients into two groups of 2,725 and 2,885 cases to be used for the discovery phase and for the validation phase, respectively. For controls, we included 2,331 and 3,395 females consisting of healthy volunteers from Midosuji Rotary Club, Osaka, Japan and Health Science Research Resource Bank as well as individuals in the Biobank who had no history of cancer as controls for discovery and validation phases, respectively. The demographic data of patients recruited for this study is summarized in Table 1. All individuals who participated in this study provided written inform consent. This study was approved by the ethical committees of the Institute of Medical Sciences, the University of Tokyo and RIKEN Center for Integrative Medical Sciences.

**Table 1:** Demographic data of patients recruited for this study.

	GWAS Set	Validation Set
**Case**	2642	2885
Age	56.9	59.8
**Menopause status**		
Postmenopausal	2088	2201
Premenopausal	99	184
Unknown	455	500
**Family history**		
Yes*	305	365
No	2337	2520
**Estrogen Receptor**		
Positive	1146	1459
Negative	663	378
**Progesterone Receptor**		
Positive	949	1207
Negative	824	574
**HER2 Receptor**		
0	372	465
+1	324	307
+2	185	151
+3	176	105
No Staining Information	354	441
	**GWAS Set**	**Validation Set**
**Control**	2099	3395
Age	56.0	44.4
**Menopause status**		
Postmenopausal	1511	1377
Premenopausal	13	231
Unknown	807	1787

* Family members who have breast and/or ovarian cance.

### Genotyping and quality control

For the GWAS discovery stage, we genotyped both case and control samples using Illumina OmniExpress BeadChip that contained a total of 733,202 SNPs. After a standard SNP quality control which excluded SNPs with call rate of <0.98, those that deviated from the Hardy-Weinberg equilibrium (P≤1.0×10^−6^), those on the X chromosome and non-polymorphic SNPs, a total of 550,026 SNPs were used for further analysis. The cluster plot of 100 SNPs that revealed the strongest associations were checked by visual observation to exclude SNPs with ambiguous genotypes. For sample quality control, we evaluated cryptic relatedness for each sample with identity-by-state method. To examine population stratification of this study, we performed principal component analysis (PCA) using EIGENSTRAT software v2.0 (http://genepath.med.harvard.edu/~reich/Software.htm) with four reference populations from the HapMap data as reference including Europeans (represented by Caucasian from UTAH, CEU), Africans (represented by Yoruba from Ibadan, YRI) and East Asians (represented by Japanese from Tokyo, JPT, and Han Chinese from Beijing, CHB) (Figure S1a). We plotted the scatter plot by using the top two associated principal components (eigenvectors) to identify outliers who did not belong to the JPT/CHB cluster. Subsequently, we performed PCA analysis using only the genotype information of the case and control subjects to further evaluate the population substructure (Figure S1b). Quantile-quantile (Q-Q) plot was constructed using observed *P*-values against expected *P*-values and an inflation factor value (λ-value) that was calculated to assess potential population stratification of the study subjects (Figure S2a and S2b). After performing PCA, we selected 2,642 cases and 2,099 controls within the major Japanese (Hondo) cluster for subsequent analysis (Figure S1b).

### Imputation analysis

To increase the power and coverage of the genome-wide association scan, we performed whole genome imputation using 1000G of East Asian population (Japanese in Tokyo JPT, Chinese in Beijing CHB and Chinese in Denver CHD) Phase I Integrated Release Version 2 dataset as reference panel to infer missing genotypes. Briefly, we prepared the input files after quality control, which excluded SNPs with genotyping rate of <98%, those that deviated from HWE (HWE *P*≤1.0×10^−6^) and those with MAF of <0.01. We then confirmed that the allele frequencies of the reference allele are comparable between the GWAS dataset and the reference panel with differences of <0.15. By using MACH1.0 (http://www.sph.umich.edu/csg/abecasis/MACH/index.html), we performed haplotype phasing with the samples' genotypes referring 1000G reference panel, estimated the map crossover and error rates using 20 iterations of the Markov chain. Subsequently, we imputed the missing genotypes using Minimac (http://genome.sph.umich.edu/wiki/Minimac). We utilized stringent imputation quality control by excluding SNPs with r^2^ value of <0.9.

### Validation study

After evaluating the associations from GWAS and whole genome imputation, we selected a total of 13 candidate loci that showed suggestive association (*P*<1.0×10^−5^) with breast cancer risk for further validation by an independent set of 2,885 cases and 3,395 controls. We genotyped the cases with the multiplex-PCR Invader assay [36] and the control samples with either Illumina OmniExpress BeadChip Kits or by imputation. To verify the accuracy of the imputation analysis, we also included surrogate SNPs that showed close link (r^2^>0.8 and D' = 1.00) to the imputed SNPs and were included in the genotype platform. Considering multiple testing at this validation stage, we applied Bonferroni significance threshold at *P*<3.85×10^−3^ (0.05/13 independent tests).

### Evaluation of previously reported loci

To verify previously-reported loci showing the association with breast cancer in the European and East Asian populations, we evaluated 67 loci in the current Japanese GWAS dataset [18]–[34]. Among the 67 SNPs examined, 6 SNPs are not polymorphic in the Japanese population, 26 SNPs are same as the previously-reported SNPs, 33 and 2 SNPs are SNPs having r^2^-value of more than 0.8 and 0.7 to the previously-reported SNPs, respectively (Table S4).

### Statistical Analysis

The case-control associations of the GWAS discovery set and validation set were evaluated using logistic regression analysis after considering age as confounding factor from PLINK software (http://pngu.mgh.harvard.edu/~purcell/plink/). The associations of the imputed SNPs were generated with mach2dat software which utilized the output results from Minimac (dosage of the imputed SNP). To have an overview of the association of SNPs with breast cancer, a Manhattan plot of the study was plotted using Haploview 4.1. Meta-analysis for the combined analysis of the discovery and validation phase was performed using inverse-variance method and heterogeneity between the two phases was evaluated using Cochran's Q test. Regional association plots were generated using Locus Zoom (http://csg.sph.umich.edu/locuszoom/).

### Weighted genetic risk score (wGRS)

To evaluate the cumulative effects of genetic variants associated with breast cancer risk, we conducted weighted genetic risk score (wGRS) analysis on the basis of genotypes of five SNPs, three significant SNPs (rs2981578 of 10q26/*FGFR2*, rs3803662 and rs12922061 of 16q12/*TOX3*) from this study and two SNPs (rs6557161 of 6q25/*ESR1* and rs10509168 of 10q21/*ZNF365*) that were reported to be associated with breast cancer risk in East Asian population and indicated suggestive association in this study. The wGRS model was developed by logistic regression analysis by incorporating five associated-SNPs and age (as covariates) using GWAS dataset to obtain the estimates (weight) of each corresponding SNP. This model was subsequently validated in an independent samples dataset drawn from the validation phase of this study. The cumulative genetic risk scores were determined by multiplying the number of risk alleles (0/1/2) of an individual by its corresponding weight, and subsequently the sum across the total number of SNPs were taken into consideration. We then classified the genetic risk score into five different categories created from the mean and standard deviation (SD); group 1, < mean-1SD; group 2, mean-1SD to mean; group 3, mean to mean+1SD; group 4, mean+1SD to mean+2SD, group 5, > mean+2SD. Odds ratio and 95% confidence interval were calculated using group 1 as a reference.

## Results

**Figure 1:**
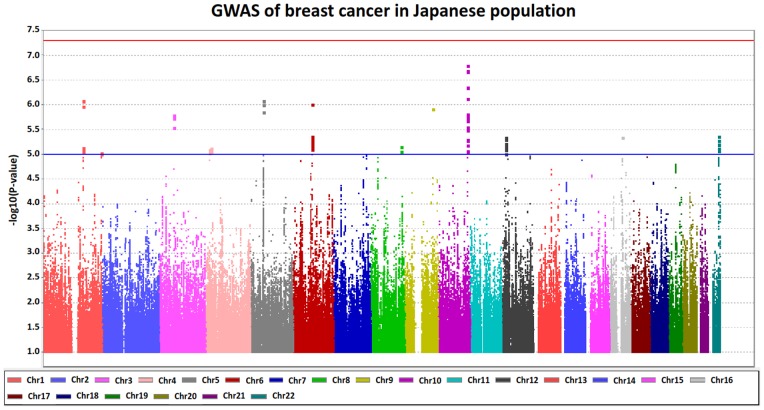
The Manhattan plot for GWAS of breast cancer in the Japanese population. This plot is based on –log_10_ (*P*-value) from GWAS and imputation analysis against chromosome position, each color represents different chromosome. Blue line indicate suggestive association threshold, *P* = 1×10^−5^ while red line indicate genome-wide significant threshold *P*<5×10^−8^.

In this study, we genotyped a total of 2,725 cases and 2,311 controls with Illumina OmniExpress BeadChip Kits that contained 733,202 SNPs to identify genetic variants associated with the susceptibility to breast cancer in the Japanese population. After quality check of the SNP genotyping data, a total of 550,026 autosomal SNPs were examined for the association by logistic regression analysis. Quantile-quantile (Q-Q) plot and the genomic inflation factor (λ) of the test statistic of this GWAS based on 550, 026 SNPs with all samples was 1.183 suggesting the existence of some population substructure (Figure S2a). To exclude the possibility of population substructure for our sample population, we performed principal component analysis (PCA). Although all the subjects participating in this study were clustered in the Asian population, there was a small portion of samples that were separated from the major Japanese (Hondo) cluster when PCA analysis was performed using only the genotype information of the case and control in the study (Figure S1a and S1b). We then used samples from the major Japanese (Hondo) cluster consisting of 2,642 cases and 2,099 controls, and found that the λ-value improved to 1.027 (Figure S2b). Hence, subsequent analysis was carried out using only samples from the major Japanese cluster. Whole genome imputation utilizing 1000G database as reference panel successfully estimated 7,791,127 SNPs. After stringent quality control by excluding SNPs with r^2^-value of <0.9, the total number of SNPs that were taken into account was 5,335,291. The Manhattan plot, plotting –log_10_ (*P*-value) from the GWAS and imputation analysis against the chromosome position, showed that there were no genetic loci achieving genome-wide significance with the threshold *P*-value of <5×10^−8^ (Figure 1).

To identify additional susceptible loci associated with breast cancer, we conducted a validation study of 13 genetic loci showing suggestive association (*P<*1×10^−5^) with breast cancer after excluding SNPs that showed linkage disequilibrium (LD) coefficient (r^2^) of >0.8 within each LD block by examining an independent set of 2,885 breast cancer cases and 3,395 controls. Among the 13 loci tested, three SNPs (rs2981578 on chromosome 10q26.13 and rs3803662 along with rs12922061 on chromosome 16q12.1) were successfully validated with Bonferroni-corrected *P*-value of <3.85×10^−3^ (0.05/13 independent tests) as shown in Table 2 and Table S1. Inverse variance meta-analysis indicated that these three SNPs surpass genome-wide significance level (*P*-value<5×10^−8^) after combining the GWAS and the validation study with no significant heterogeneity (*P*-value >0.05) between the two stages (Table 2).

**Table 2:** Association study of SNPs on chromosome 10q26.13 and 16q12.1.

CHR	SNP	BP	Stage	RA	NRA	NCASES	NCONTROLS	RAF_Case	RAF_Ctrl	P_value	OR	SE	L95	U95	P_hetero	*Gene*	rel.loci
10	rs2981578	123340311	GWAS	C	T	2642	2097	0.571	0.517	2.25E-07	1.238	0.041	1.142	1.342		*FGFR2*	0
10	rs2981578	123340311	Rep	C	T	2883	3395	0.556	0.512	1.63E-06	1.213	0.040	1.121	1.313			
10	rs2981578	123340311	Combined	C	T	5525	5492	0.563	0.514	1.31E-12	1.225	0.028	1.158	1.296	7.18E-01		
16	rs12922061	52635000	GWAS	T	C	2641	2099	0.287	0.245	4.50E-06	1.244	0.048	1.133	1.365		*LOC643714*	0
16	rs12922061	52635000	Rep	T	C	2880	3395	0.278	0.239	1.41E-05	1.219	0.046	1.115	1.333			
16	rs12922061	52635000	Combined	T	C	5521	5494	0.282	0.241	3.97E-10	1.231	0.032	1.153	1.314	7.60E-01		
Another SNP on 16p12 that independently associated with breast cancer																	
16	rs3803662	52586341	GWAS	T	C	2642	2097	0.570	0.531	9.09E-05	1.178	0.042	1.085	1.279		*LOC643714*	0
16	rs3803662	52586341	Rep	T	C	2880	3392	0.572	0.517	4.69E-08	1.245	0.040	1.151	1.347			
16	rs3803662	52586341	Combined	T	C	5522	5489	0.571	0.522	2.79E-11	1.213	0.029	1.146	1.284	3.40E-01		

CHR: chromosome, SNP: single nucleotide polymorphism, BP: SNP genomic location, RA: Risk allele, NRA: Non-risk allele, NCASES: Number of cases, NCONTROLS: Number of controls, RAF: risk allele frequency, P_value: *P*-value from logistic regression analysis after age adjustment, OR: odds ratio, L95: lower 95% confidence interval, U95: upper 95% confidence interval, P_hetero: heterogeneity test with Cochran Q-test, rel.loci: distance of the SNP from the gene, GWAS: genome-wide association study, Rep: validation study.

**Figure 2:**
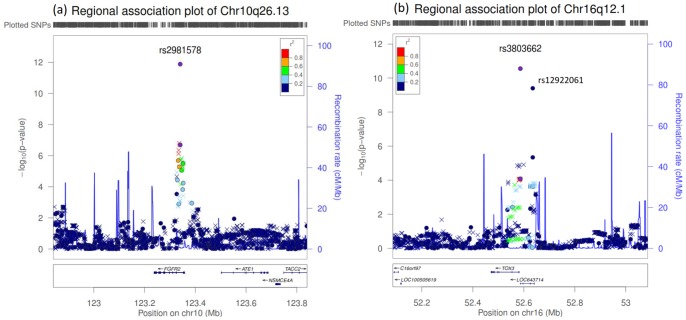
Regional association plots for two significantly associated loci with breast cancer in Japanese population, (a) chromosome 10q26.13 (*FGFR2*) and (b) chromosome 16q21.1 (*TOX3-LOC643714*). SNPs from the GWAS are plotted as circles; imputed SNPs are plotted as crosses. The color intensity reflects the extent of LD with the marker SNP: red, (r^2^≥ 0.8), orange (0.6≤r^2^≤0.8), green (0.4≤r^2^≤0.6), light blue (0.2≤r^2^≤0.4) and dark blue (r^2^<0.2). Purplish blue lines represent local recombination rates. The SNP position is based on NCBI build 37.

The most significantly associated SNP, rs2981578 (combined *P*-value of 1.31×10^−12^, OR = 1.23; 95% CI = 1.16–.30), is located within the second intron of the *FGFR2* gene on chromosome 10q26.13 (Table 2 and Figure 2a). Variants on this gene have been the most frequently validated to be associated with breast cancer in multiple populations. For chromosome 16q21.1, we successfully validated rs12922061 (combined *P*-value of 3.97×10^−10^, OR = 1.23; 95% CI = 1.15–.31) to be significantly associated with breast cancer (Table 2 and Figure 2b). After conditioning the effect of rs12922061, rs3803662 remained suggestively associated and was successfully validated after additional samples with a combined *P*-value of 2.79×10^−11^ (OR = 1.21; 95% CI = 1.14–.25). Two of these SNPs remained significant (*P*-value<0.0001) after performing condition analysis by using one of the SNP as covariate, suggesting the independency of association with breast cancer (Table S2). Additionally, the r^2^ value between these two SNPs is only 0.17, indicating they are not closely linked with each other. Haplotype analysis of the two SNPs did not reveal stronger association than a single SNP association after 100,000 permutation analysis (Table S3). The SNP, rs3803662, is located in the last exon of *LOC643714* and near to the 5′ end of *TOX3*; whilst rs12922061 is located in the first intron of *LOC643714.*

In addition to perform GWAS for breast cancer in Japanese population, we also evaluated the association of previously-reported breast cancer risk loci in the European and East Asian populations. We evaluated a total of 61 SNPs after excluding 6 SNPs that are not polymorphic in Japanese population (Table S4). Among the 61 SNPs, eight SNPs (rs4415084 of 5p12/*MRPS30*, rs6557161 of 6q25/*ESR1*, rs7465364 of 8p21/*RPL17p33*, rs672888 of 8q24/*MYC*, rs10509168 of 10q21/*ZNF365*, rs1219648 of 10q26/*FGFR2*, rs17221259 of 12p13/*ATF7IP* and rs3803662 of 16q12/*TOX3*) showed suggestive association (*P*-value<0.05) with breast cancer in Japanese population (Table 3). All of these suggestively-associated SNPs possessed the same risk allele and showed the same direction of association that was indicated in the previous reports.

**Table 3:** Association of previously reported to be breast cancer susceptibility loci in current Japanese GWAS dataset.

CHR	SNP	Chr.loci/Gene	BP	Risk allele	Ref. allele	Case_N	Ctrl_N	RAF_Case	RAF_Ctrl	P-value	OR	SE	L95	U95	Remarks	Reference
5	rs4415084	5p12/*MRPS30*	44662515	T	C	2642	2098	0.601	0.573	8.68E-03	1.118	0.043	1.029	1.215		[27]
6	rs6557161	6q25/*ESR1*	151950235	G	A	2642	2099	0.316	0.286	1.17E-03	1.160	0.046	1.061	1.269	r^2^ = 1.000 with rs2046210	[22]
8	rs7465364	8p21/*RPL17P33*	29505165	A	G	2641	2099	0.344	0.325	4.91E-02	1.091	0.044	1.000	1.191	r^2^ = 0.961 with rs9693444	[32]
8	rs672888	8q24/*MYC*	128345463	G	A	2642	2099	0.540	0.516	1.97E-02	1.102	0.042	1.016	1.195	r^2^ = 0.858 with rs13281615	[18]
10	rs10509168	10q21/*ZNF365*	64257828	G	A	2642	2099	0.484	0.461	2.49E-02	1.099	0.042	1.012	1.193	r^2^ = 0.863 with rs10822013	[28]
10	rs1219648	10q26/*FGFR2*	123346190	C	T	2641	2099	0.405	0.360	9.85E-06	1.208	0.043	1.111	1.314		[18], [19], [23], [25], [33], [34]
12	rs17221259	12p13/*ATF7IP*	14410485	G	A	2641	2099	0.243	0.204	6.68E-06	1.252	0.050	1.136	1.381	r^2^ = 0.744 with rs12422552	[32]
16	rs3803662	16q12/*TOX3*	52586341	T	C	2642	2097	0.570	0.531	9.09E-05	1.178	0.042	1.085	1.279		[18], [20], [23], [25], [27]

CHR: chromosome, SNP: single nucleotide polymorphism, Chr.loci/Gene: Chromosome location/Gene, BP: SNP genomic location, Ref: reference, Case_N: Number of cases, Ctrl_N: Number of controls, RAF: risk allele frequency, P_value: *P*-value from logistic regression analysis after age adjustment, OR: odds ratio, L95: lower 95% confidence interval, U95: upper 95% confidence interval.

After developing wGRS model using five SNPs from the GWAS dataset, the model was subsequently validated in an independent sample set represented by the validation samples. The cumulative effect of five SNPs evaluated by the wGRS analysis indicated that odds ratio of each category increased according to the level of risk score, and individuals who are in category five carrying the most risk alleles have 2.2 times higher risk to develop breast cancer when utilizing category 1 as a reference (Table 4).

**Table 4:** wGRS using 5 significant associated SNPs evaluated on independent validation sample set.

Category	Case (N = 2869)	Control (N = 3385)	%_Case	%_Ctrl	OR	95%_CI
1	386	676	0.135	0.200	REF	
2	937	1203	0.327	0.355	1.364	1.172–.587
3	998	1056	0.348	0.312	1.655	1.422–.927
4	464	382	0.162	0.113	2.127	1.769–.558
5	84	68	0.029	0.020	2.163	1.535–.050

## Discussion

To investigate the involvement of common genetic variants (SNPs) associated with breast cancer in the Japanese population, we performed GWAS, whole genome imputation using 1000G database as reference panel and validation study using a total of 5,527 breast cancer cases and 5,494 controls individuals. We successfully validated the association of chromosome 10q26.13 (*FGFR2*), and 16q12.1 (*TOX3*-*LOC643714*). In addition to the two aforementioned loci, we validated a total of 67 loci that were previously reported the association with breast cancer and identified six additional loci (rs4415084 of 5p12/*MRPS30*, rs6557161 of 6q25/*ESR1*, rs7465364 of 8p21/*RPL17p33*, rs672888 of 8q24/*MYC*, rs10509168 of 10q21/*ZNF365* and rs17221259 of 12p13/*ATF7IP*) to have suggestive association (*P<*0.05) with breast cancer in Japanese population. Further fine mapping of these loci might identify insightful findings for future analysis.

Hunter DJ *et al*. first reported the association of *FGFR2* with breast cancer in 2007 [19]. Since then, this locus has been successfully validated in various populations throughout the world including those of European ancestry, Asian, Ashkenazi Jewish and Israeli populations [18], [37]–[39]. *FGFR2* encodes fibroblast growth factor receptor type 2, which is a receptor tyrosine kinase playing a critical role in the growth signaling pathway that is involved in growth and differentiation of cells in various tissues including the breast and kidney [40], [41]. All the SNPs that were found to be associated with breast cancer are located in intron 2 of the gene; the risk allele of rs2981578, a SNP that was identified in this study, created a putative binding site for Oct-1/Runx2, which gives rise to a strong protein-DNA complex that alters binding of the transcription factor and causes differential expression between the common and minor haplotypes of *FGFR2* [42]. Additionally, Zhu et al. also reported that there is a potential role of histone 3/4 acetylation in modulating access to the polymorphic sites within intron 2 in addition to downstream splicing sites in generating variable FGFR2 levels and isoforms in breast cancer [43].

The second significantly associated locus is located on chromosome 16q12.1 (*TOX3*-*LOC643714*). *LOC643714* is an uncharacterized gene of unknown function; *TOX3*, also known as *TNRC9* or *CAGF9*, encodes a high mobility group box nuclear protein, which is involved in regulating calcium-dependent transcription [44]. A previous study indicated that increased expression of TOX3 could be a predictor of breast cancer metastasis to bone [45]. In this study, we identified two independently associated SNPs, rs3803662 and rs12922061, with breast cancer in the Japanese population. The minor allele of rs3803662 is reported to cause lower mRNA expression of *TOX3* gene, and this regulatory SNP may alter the expression of a distant gene, *RBL2*, in *cis* [46].

Although wGRS of five associated loci with breast cancer in the Japanese population revealed that individuals with the highest risk (category 5) have 2.2 times higher risk than those with the lowest risk (category 1), it is believed that a complex disease such as breast cancer would be affected by a large number of common genetic variants that have very modest effects. This phenomenon was also supported by the six additional reported loci that showed suggestive association in this dataset, indicating that our current dataset is still under statistical power. Hence, to increase the power and to enlarge the sample number, there is a need for more local and international institutions to collaborate with each other in identifying more common variants associated with breast cancer, which hopefully will lead to the development of promising and accurate prediction system.

## Supporting Information

Figure S1 Principal component analysis of (a) Case and control samples of this study with four reference populations from the HapMap database which include Europeans (represented by Caucasian from UTAH, CEU), Africans (represented by Yoruba from Ibadan, YRI) and East Asians (represented by Japanese from Tokyo, JPT, and Han Chinese from Beijing, CHB). (b) Case and control samples of this study. Samples from the major cluster (within the black oval circle) were selected for further analysis.
(TIFF)

Figure S2 Quantile-quantile (Q-Q) plot for GWAS of breast cancer in Japanese population with (a) All samples (λ = 1.18) and (b) Major Japanese (Hondo) cluster (λ = 1.03).
(TIFF)

Table S1 Association study of the 13 selected loci.
(XLS)

Table S2 Conditioning analysis of SNPs on chromosome 16q12.1.
(XLS)

Table S3 Haplotype analysis and association of SNPs on chromosome 16q12.1.
(XLS)

Table S4 Association study of previously reported breast cancer associated loci.
(XLS)
