# Supplementary figures and images for: Genome-Wide Association Study of Breast Cancer in the Japanese Population
Source: PLoS One. 2013 Oct 15;8(10):e76463. doi: 10.1371/journal.pone.0076463 (PMC3797071; doi:10.1371/journal.pone.0076463)

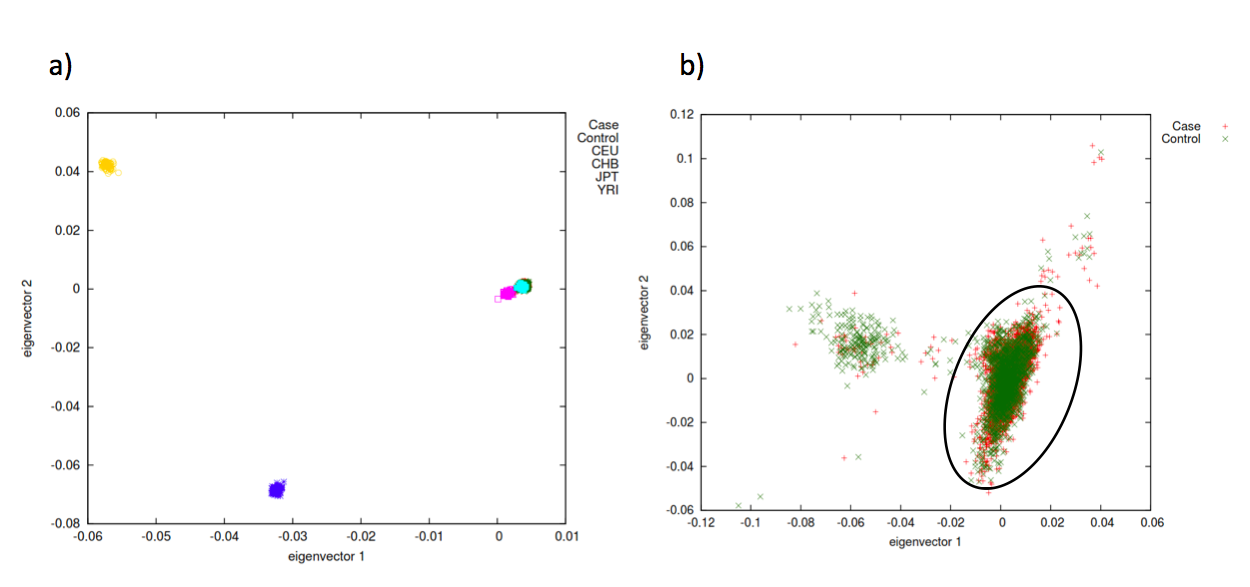

Supplement: Figure S1 — Principal component analysis of (a) Case and control samples of this study with four reference populations from the HapMap database which include Europeans (represented by Caucasian from UTAH, CEU), Africans (represented by Yoruba from Ibadan, YRI) and East Asians (represented by Japanese from Tokyo, JPT, and Han Chinese from Beijing, CHB). (b) Case and control samples of this study. Samples from the major cluster (within the black oval circle) were selected for further analysis. (TIFF) [file pone.0076463.s001.tiff]

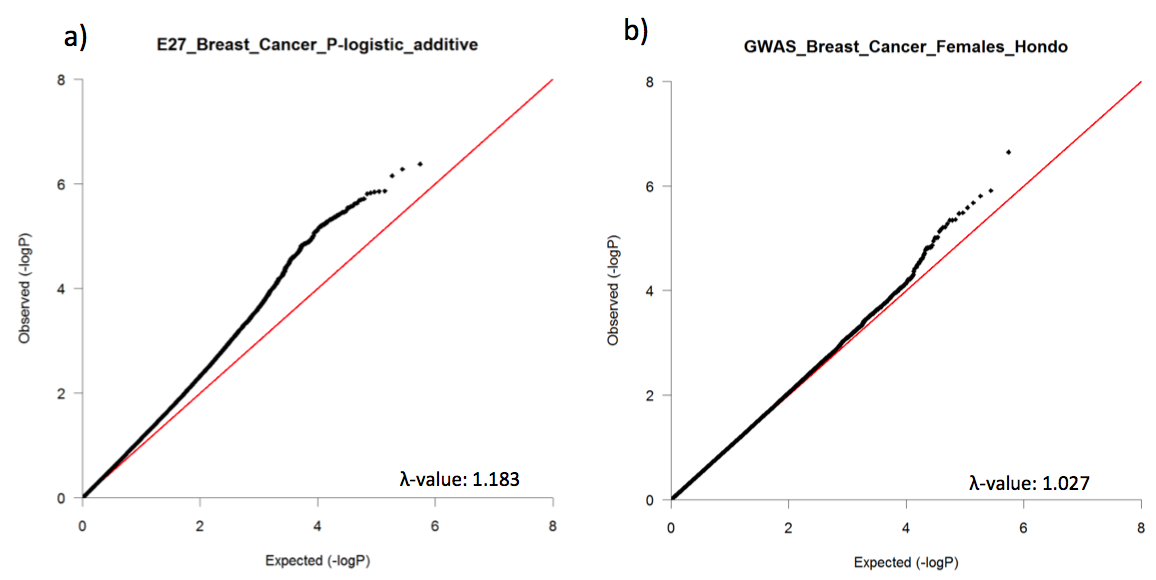

Supplement: Figure S2 — Quantile-quantile (Q-Q) plot for GWAS of breast cancer in Japanese population with (a) All samples (λ = 1.18) and (b) Major Japanese (Hondo) cluster (λ = 1.03). (TIFF) [file pone.0076463.s002.tiff]
